# ATR Inhibition in Advanced Urothelial Carcinoma

**DOI:** 10.1016/j.clgc.2022.10.016

**Published:** 2022-11-04

**Authors:** Ryan C. Leibrandt, Mei-Juan Tu, Ai-Ming Yu, Primo N. Lara, Mamta Parikh

**Affiliations:** a University of California at Davis School of Medicine, Department of Internal Medicine, Division of Hematology Oncology, Sacramento, California, United States of America; b University of California at Davis School of Medicine, Department of Biochemistry and Molecular Medicine, Sacramento, California, United States of America; c University of California at Davis Comprehensive Cancer Center, University of California at Davis School of Medicine, Department of Internal Medicine, Division of Hematology Oncology, Sacramento, California, United States of America

## Abstract

The ataxia telangiectasia and Rad3-related (ATR) checkpoint kinase 1 (CHK1) pathway is intricately involved in protecting the integrity of the human genome by suppressing replication stress and repairing DNA damage. ATR is a promising therapeutic target in cancer cells because its inhibition could lead to an accumulation of damaged DNA preventing further replication and division. ATR inhibition is being studied in multiple types of cancer, including advanced urothelial carcinoma where there remains an unmet need for novel therapies to improve outcomes. Herein, we review preclinical and clinical data evaluating 4 ATR inhibitors as monotherapy or in combination with chemotherapy. The scope of this review is focused on contemporary studies evaluating the application of this novel therapy in advanced urothelial carcinoma.

## Introduction

Bladder cancer is the sixth most common malignancy in the United States with an annual incidence of approximately 80,000 cases and 17,000 deaths.^1^ The 5-year relative survival rate for locally advanced or metastatic disease remains around 15% despite current systemic therapy regimens.^1 , 2^ The current standard of care in treatment-naïve patients is cisplatin-based chemotherapy combinations with an overall response rate approaching 45%.^3 , 4^ The median age at diagnosis of bladder cancer is 73-years-old, therefore precluding many patients from receiving platinum-based chemotherapy due to poor performance status, underlying co-morbidities or renal impairment.^5^ Newer treatments, such as immune checkpoint inhibitors, targeted therapy for those with FGFR gene alterations, and antibody-drug conjugates, have led to improvements in survival among patients with advanced urothelial carcinoma.^5^ Nevertheless, the overall poor prognosis underlies the importance of developing novel treatments in this field. The objective of this article is to review contemporary data regarding ATR inhibitors and their clinical application in bladder cancer to date.

## DNA Damage Response

**Figure 1:**
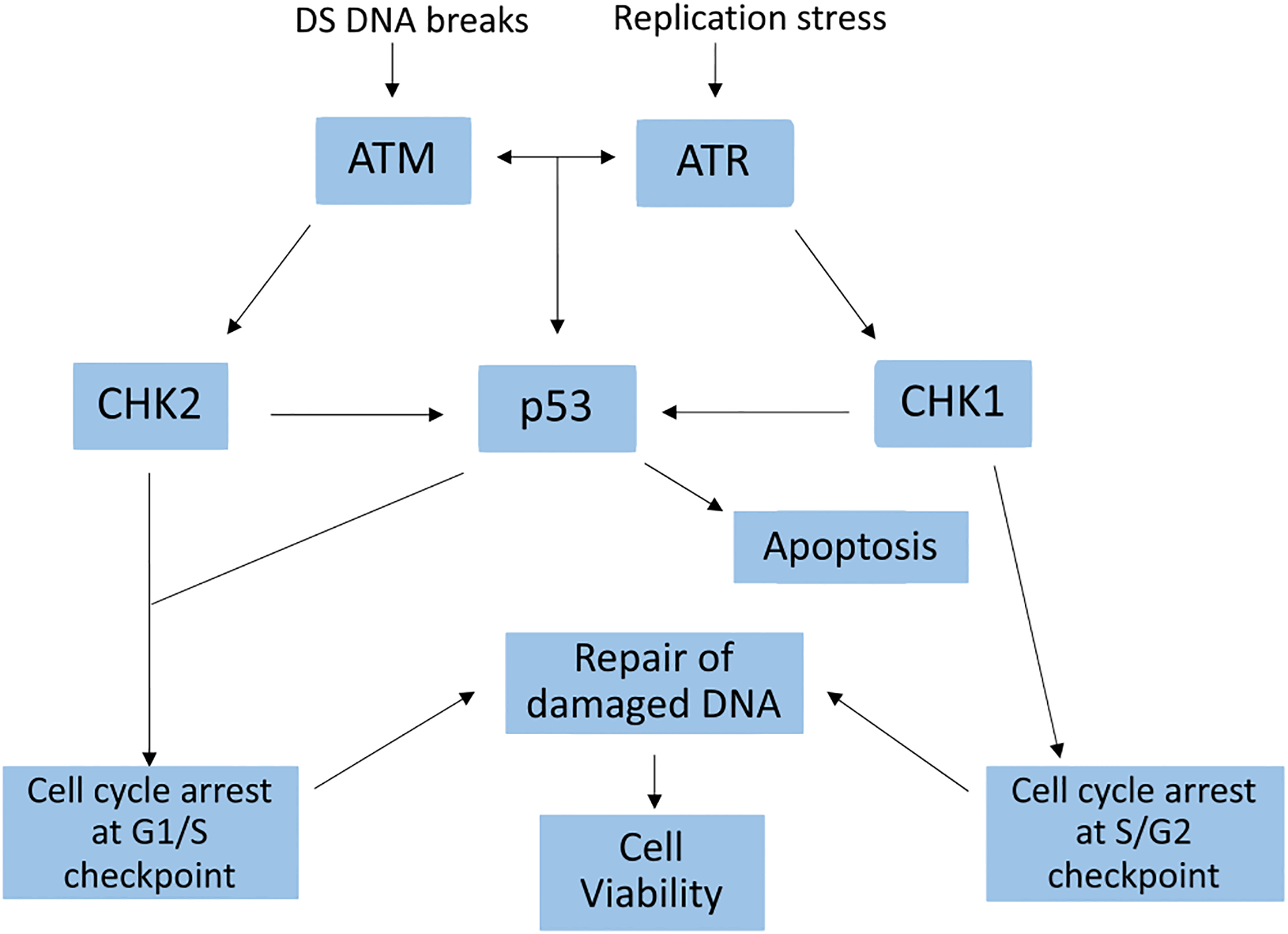
ATM and ATR in the DNA damage response system

One target of interest in bladder cancer is the DNA-damage response (DDR) system, a network of inter-connected signaling pathways which detects and repairs DNA damage, thus promoting cell survival.^6–8^ Ataxia telangiectasia mutated (ATM) and ataxia telangiectasia and Rad3-related (ATR) are members of the phosphatidylinositol 3-kinase-related kinase (PI3K) family of serine/threonine protein kinases which play an integral role in DDR. Their dual function is to preserve genomic integrity in the face of endogenous or exogenous insults such as chemotherapy or ionizing radiation.^6 , 9 , 10^ ATM recognizes double-stranded DNA breaks (DSB) and activates its effector kinase, CHK2, leading to a sequence of events causing cell cycle arrest at the G1/S phase checkpoint.^11^ ATM coats single-stranded DNA (ssDNA) with replication protein A (RPA) which is then recognized by ATR.^10 , 12–16^ Once activated as a result of DNA replication-associated stress, ATR phosphorylates its effector kinase, CHK1, resulting in a multitude of downstream events causing cell cycle arrest at the S/G2 checkpoint, thus enabling repair of damaged DNA^6 , 15 - 17^ (Figure 1). A variety of cancer cell types are deficient in ATM, forcing them to upregulate ATR to maintain cell viability.^6 , 15 , 16^ ATR inhibition in ATM-deficient cell lines results in synthetic lethality and renders these tumors more susceptible to DNA-damaging chemotherapies.^18^

ATM mutations have been identified in patients with bladder cancer and are associated with inferior overall survival.^19^ A retrospective analysis of patients with relapsed or advanced urothelial carcinoma who had undergone genomic sequencing or were selected from The Cancer Genome Atlas bladder cohort found nearly 15% with ATM alterations, and that these alterations correlated with shorter survival.^20^ A possible explanation for their presence is the strong association between cigarette smoking and the increased risk of bladder cancer. One mechanism by which tobacco exerts its carcinogenic effects is by increasing tumor mutational burden which is often associated with errors in DDR.^21 , 22^ This feature of bladder cancer makes it a potential target for ATR inhibition. ATM mutational status may also represent a promising biomarker for the selection of appropriate patients for targeted treatment.

## Synergy of ATR Inhibitors and Cisplatin

Cisplatin exerts its cytotoxic effects via DNA cross-links which ultimately inhibit DNA synthesis and cause cell cycle arrest at the G2/M checkpoint.^23^ In turn, cancer cells appear to upregulate ATR transiently, a potential means of developing resistance to chemotherapy.^24^ Therefore, combining cisplatin with ATR inhibition could represent a way to subvert this resistance mechanism and enhance cytotoxicity.

ATR inhibitors have demonstrated synergy with cisplatin in preclinical studies. For example, ceralasertib resulted in prolonged cytotoxicity of cisplatin in ATM-deficient NSCLC cell lines.^25^ Even in cell lines with intact ATM signaling, the combination of cerale-sertib with cisplatin potentiates cytotoxicity.^25^ Similarly, berzosertib in combination with cisplatin demonstrated enhanced cytotoxicity both in lung cancer cell lines in vitro and in vivo in patient-derived primary lung xenografts.^26^ In 3 cisplatin-insensitive NSCLC models, berzosertib with cisplatin resulted in complete inhibition of tumor growth.

At our institution, we have demonstrated synergistic effects between elimusertib and platinum-based drugs in human bladder cancer cell lines (Figure 3 A–F) as well as lung cancer cell lines. Bladder cancer cell lines (5637 and T24) were treated with cisplatin, carboplatin, elimusertib or the combination of cisplatin/carboplatin and elimusertib at a series of concentrations. MTT assay was used to determine cell viability 72 hours after the treatments. Combination effects were determined by Combination Index (CI) method in which CI < 1 indicates the presence of synergism. Both cisplatin and carboplatin exhibit strong synergism when combined with elimusertib in the control of bladder cancer cell growth (C and F). The combination also demonstrated increased inhibition of cell viability compared to either single agent, as evidenced by comparing the second data point across all groups (A, B, D, E). Taken together, there is significant in vitro and in vivo evidence that combination therapy has a potential therapeutic benefit in patients.

## ATR Inhibitors

**Figure 2:**
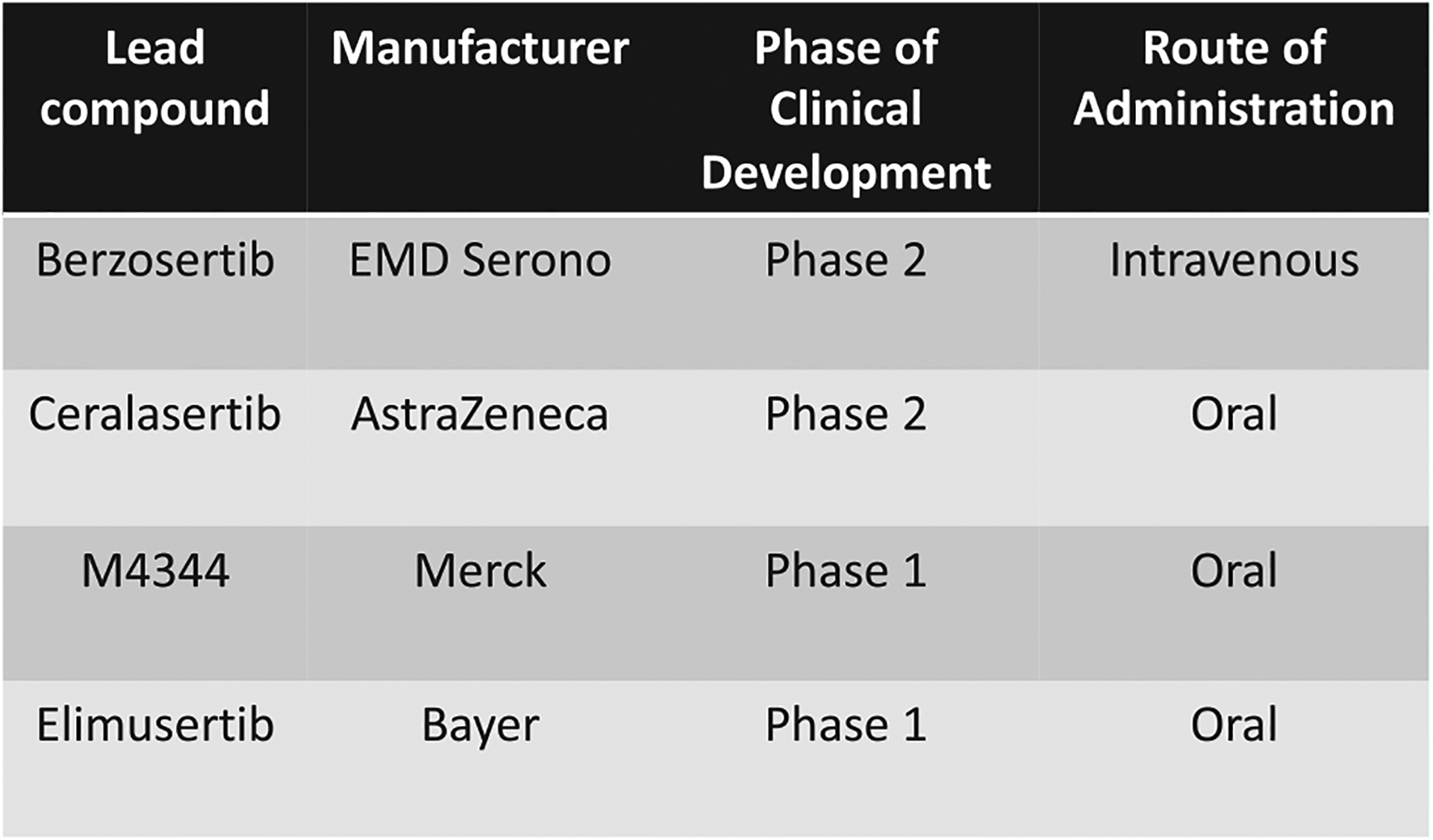
ATR inhibitors under clinical development

**Figure 3:**
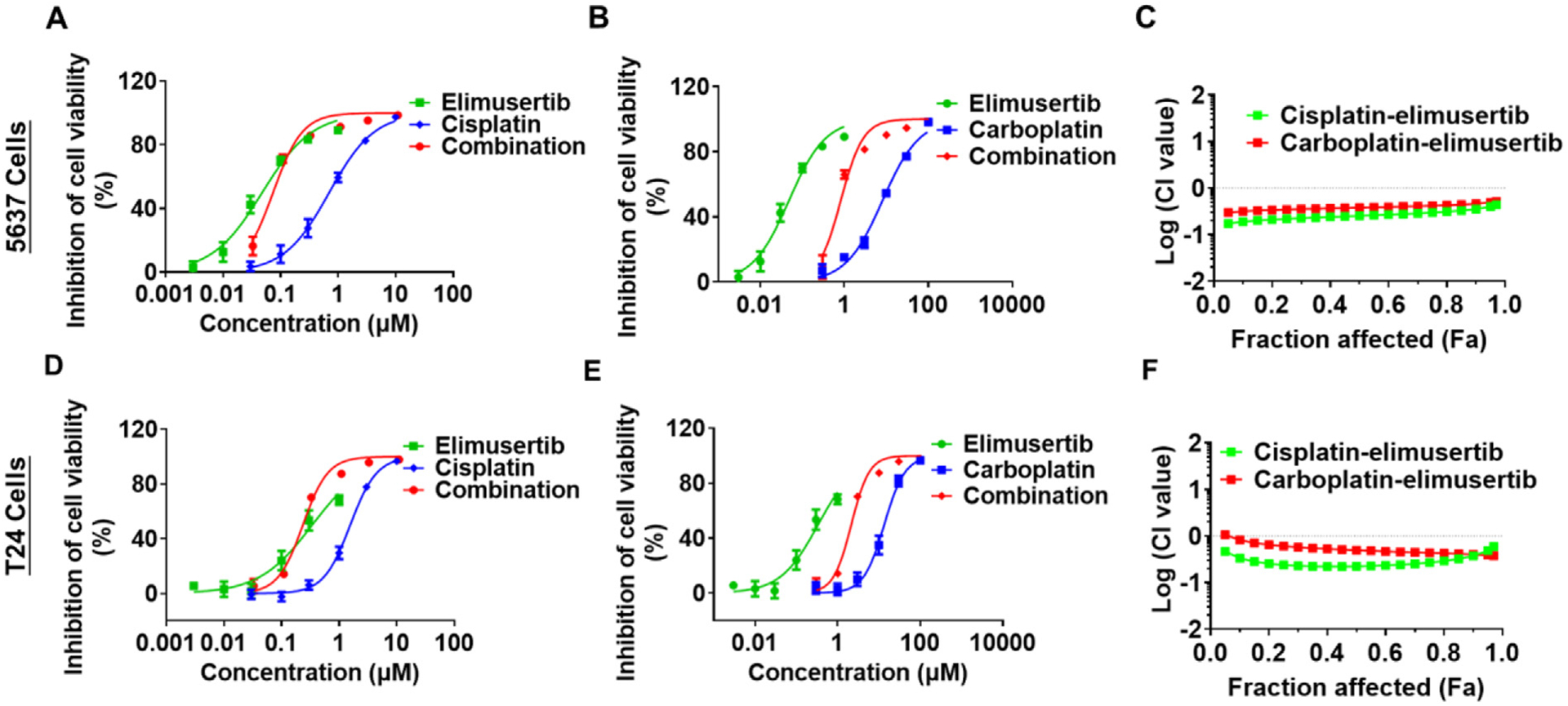
Combination of elimusertib and platinum chemotherapy synergistically inhibits the proliferation of human bladder cancer cells. Human bladder cancer 5637 (A-C) and T24 (D-F) cells were treated with various concentrations of elimusertib alone, cisplatin or carboplatin alone, or the combination of elimusertib and cisplatin/carboplatin for 72 h, and cell viability then was determined by MTT assay. Elimusertib alone, cisplatin (A & D) or carboplatin (B & E) alone, and the combination of elimusertib and cisplatin/carboplatin exhibited dose-dependent effects in the inhibition of bladder cancer cell viability. Combination treatments showed stronger efficacy than the corresponding single drug treatments in both cell lines. Values are mean ±SD (N = 5/group). (C & F) Combination index (CI) versus Fraction affected (Fa) plots revealed a synergism for the combination of elimusertib and cisplatin/carboplatin in suppressing bladder cancer cell viability

There are several ATR inhibitors in development, but this review will focus on those that have progressed to early-phase clinical trials (Figure 2). These include berzosertib (formerly M6220 and VX-970, EMD Serono), ceralasertib (formerly AZD6738, AstraZeneca), M4344 (formerly VX-803, Merck), and elimusertib (formerly BAY1895344, Bayer).

Berzosertib is a first-in-class intravenously administered ATR inhibitor with *in vitro* evidence of synergy with cisplatin in lung tumor xenografts, lowering the mean 50% inhibitory concentration of cisplatin by approximately 1.5-fold.^26^ Additionally, there is evidence of synergy between ATR inhibitors and gemcitabine in pancreatic cell lines.^27 , 28^ This led to a Phase 1 study of 40 patients with advanced cancer who either received berzosertib monotherapy or combination therapy with carboplatin.^29^ Although urothelial carcinoma patients were not enrolled, a large proportion of patients experienced stable disease, defined by Response Evaluation Criteria in Solid Tumors (RECIST) version 1.1, as their best response. An NCI-sponsored randomized phase 2 trial evaluated the role of berzosertib in combination with chemotherapy in metastatic urothelial carcinoma. In this study, 87 patients with treatment-naïve metastatic urothelial carcinoma were randomized to receive either gemcitabine and cisplatin or gemcitabine and cisplatin plus berzosertib. There was no difference in the primary endpoint of progression-free survival among the groups, and there was an apparent trend towards inferior overall survival in the experimental arm (14.4 vs. 19.8 months; HR 1.42; 95% CI 0.76–2.68). This is potentially explained by attenuated dosing of the chemotherapy doublet as part of the initial study design and later compounded by higher rates of grade 3 and 4 cytopenias in the experimental arm which necessitated further per-protocol chemotherapy dose reductions. These dosing adjustments ultimately resulted in a significantly lower cumulative dose of cisplatin received by patients randomized to the experimental arm (250 mg/m ^2^ vs. 370 mg/m ^2^ ; *P* < .001).^30^

Ceralasertib is an oral ATR inhibitor that has demonstrated in vivo synergy with cisplatin against NSCLC.^25^ When ATM-deficient NSCLC cell lines were treated with ceralasertib, synthetic lethality was observed. Preclinical evidence specifically in urothelial carcinoma has not been reported to date. There is an ongoing Phase 2 trial (NCT03682289) evaluating ceralasertib alone or in combination with a PARP inhibitor for different types of advanced cancer, including urothelial carcinoma. This study is unique in that one arm of the study population is limited to patients with ATM mutations, a group that may derive the most benefit from ATR inhibition. An actively recruiting Phase 1 trial (NCT02264678) of ceralasertib is also stratifying patients based on ATM mutational status, signifying its potential to serve as a biomarker.

M4344 is another oral ATR inhibitor showing synergy with DNA-damaging chemotherapy based on preclinical data.^31^ A phase 1 trial (NCT04149145) has been initiated to evaluate M4344 in combination with a PARP inhibitor for ovarian cancer. A phase 1 first-in-human trial (NCT02278250) of M4344 alone or in combination with chemotherapy for advanced solid tumors was recently completed. Results from the trial have not yet been reported.

Elimusertib is an oral, highly selective ATR inhibitor with preclinical evidence of anti-tumor activity in cell lines with DDR defects. In a Phase 1 study of elimusertib monotherapy, 45% of patients with advanced solid tumors were found to have either loss of ATM expression or an ATM deleterious mutation. This study was notable for 4 patients with ATM deficiencies experiencing partial responses with elimusertib.^32^ One patient with collecting duct renal cell carcinoma had loss of ATM expression and achieved a partial response, with the best response being −69%. Furthermore, this patient received the maximal tolerated dose (MTD) for a treatment duration of 385 days which continued at the time of the data cutoff.^33^ Adverse events leading to the MTD primarily included anemia, thrombocytopenia and neutropenia. An NCI-sponsored phase I trial (NCT04491942) is testing the addition of elimusertib to chemotherapy in advanced solid tumors. In the first cohort of the study, patients with advanced solid tumors are treated with elimusertib in combination with cisplatin in a 3 + 3 design to establish a MTD of the doublet. Once the MTD of the doublet is established, the second cohort of the study will treat patients with elimusertib in combination with cisplatin plus gemcitabine with escalating doses to establish the MTD for the triplet. The triplet MTD will be further expanded to enroll patients with advanced urothelial carcinoma in attempts to further elucidate the clinical safety and efficacy of elimusertib.

## Discussion

A limitation of many studies evaluating ATR inhibition is the inclusion of an unselected patient population. As detailed in this review, ATM deficient cancers appear susceptible to ATR inhibition and therefore could be a subgroup with pronounced benefit.^26^ In the phase 1 study of berzosertib, a patient with metastatic colorectal cancer harboring an ATM mutation experienced a complete response.^29^ In the phase I study of elimusertib, all 4 patients who achieved a partial response had ATM aberrations.^33^ This is consistent with in vitro studies demonstrating synthetic lethality of ATR inhibitors in cell lines lacking ATM function. Future studies involving ATR inhibitors could involve a selected study population of patients with ATM mutations to confirm this association.

The optimal sequence of chemotherapy and ATR inhibition requires further investigation. Based on the preclinical data from our institution, we hypothesize there is sequence specificity whereby chemotherapy followed by ATR inhibition yields greater cytotoxicity compared to the reverse sequence or concurrent therapy. This hypothesis will be further studied in the Phase 1 trial (NCT04491942) which administers cisplatin on day 1 and elimusertib on days 2 and 9 of a 21-day cycle.

Phase I studies of single agent berzosertib and elimusertib reported cytopenias as a dose-limiting-toxicity (DLT).^29 , 33^ This toxicity is compounded when combined with platinum chemotherapy which is known to cause myelosuppression. In the Phase 2 study evaluating berzosertib with gemcitabine and cisplatin, cytopenias led to patients receiving lower cumulative doses of cisplatin, likely compromising efficacy in bladder cancer.^30^ Therefore, future studies will need to consider these DLTs when combining agents with ATR inhibitors.

ATR inhibition in combination with immune checkpoint inhibition (ICI) may be a feasible approach with a lower risk of toxicity compared to chemotherapy. In prospective trials of patients with metastatic urothelial carcinoma treated with anti-PD-1/PD-L1 blockade, the presence of a DDR mutation was associated with a higher response rate, longer progression-free survival and overall survival.^34^ There is in vitro evidence that ATR inhibition decreases PD-L1 expression, sensitizing cancer cells to T cell killing.^35^ This has led to multiple clinical trials currently evaluating ATR inhibitors in combination with ICIs. A phase 1 trial (NCT04095273) is testing the combination of elimusertib and pembrolizumab in patients with advanced solid tumors, though urothelial carcinoma is not included. A phase 2 trial (NCT03334617) is evaluating ceralasertib alone or in combination with durvalumab in patients with NSCLC who have progressed on anti-PD-1/PD-L1 containing therapy. Another phase 1/2 trial (NCT02264678) is combining ceralasertib with durvalumab in patients with advanced solid tumors. Preliminary data from this study reported no DLTs in their limited sample size.^36^ Urothelial carcinoma is not explicitly mentioned as a focus in these studies but may represent a future direction for application of ATR inhibitors.

## Conclusion

ATR inhibition either as monotherapy or in combination with chemotherapy and/or immunotherapy is an active area of investigation. Four ATR inhibitors are under active investigation in early-phase clinical trials in a variety of cancer types, including advanced urothelial carcinoma. Safety and tolerability data thus far suggest cytopenias may be a limitation of the class, particularly when combined with cytotoxic chemotherapy. Based on these concerns, future studies evaluating ATR inhibitors may focus on pre-selecting by ATM mutational status given its prevalence in urothelial carcinoma to identify those most likely to benefit from therapy.
